# Impact of Trans-Resveratrol-Sulfates and -Glucuronides on Endothelial Nitric Oxide Synthase Activity, Nitric Oxide Release and Intracellular Reactive Oxygen Species

**DOI:** 10.3390/molecules191016724

**Published:** 2014-10-17

**Authors:** Angela Ladurner, Daniel Schachner, Katharina Schueller, Marc Pignitter, Elke H. Heiss, Veronika Somoza, Verena M. Dirsch

**Affiliations:** 1 Department of Pharmacognosy, University of Vienna, Althanstrasse 14, 1090 Vienna, Austria; E-Mails: angela.ladurner@univie.ac.at (A.L.); daniel.schachner@univie.ac.at (D.S.); elke.heiss@univie.ac.at (E.H.H.); 2 Department of Nutritional and Physiological Chemistry, University of Vienna, Althanstrasse 14, 1090 Vienna, Austria; E-Mails: katharina.schueller@univie.ac.at (K.S.); marc.pignitter@univie.ac.at (M.P.); veronika.somoza@univie.ac.at (V.S.)

**Keywords:** resveratrol metabolites, eNOS, NO, intracellular ROS levels, endothelial cells

## Abstract

Resveratrol (3,5,4'-trihydroxy-trans-stilbene) is a polyphenolic natural product mainly present in grape skin, berries and peanuts. In the vasculature resveratrol is thought to boost endothelial function by increasing endothelial nitric oxide synthase (eNOS) expression, by enhancing eNOS activity, and by reduction of reactive oxygen species (ROS) levels. Recent studies show that dietary resveratrol is metabolized in the liver and intestine into resveratrol-sulfate and -glucuronide derivatives questioning the relevance of multiple reported mechanistic *in vitro* data on resveratrol. In this study, we compare side by side different physiologically relevant resveratrol metabolites (resveratrol sulfates- and -glucuronides) and their parent compound in their influence on eNOS enzyme activity, endothelial NO release, and intracellular ROS levels. In contrast to resveratrol, none of the tested resveratrol metabolites elevated eNOS enzyme activity and endothelial NO release or affected intracellular ROS levels, leaving the possibility that not tested metabolites are active and able to explain *in vivo* findings.

## 1. Introduction

Endothelial nitric oxide (NO), the product of the enzyme endothelial nitric oxide synthase (eNOS), is considered a major anti-inflammatory and anti-atherogenic mediator in the cardiovascular system [1]. In particular, the impaired availability of endothelial NO, resulting in endothelial dysfunction, is regarded as the initial step in the development of cardiovascular diseases [2].

The polyphenol resveratrol (3,5,4'-trihydroxy-trans-stilbene), which is found predominantly in grape skin, red wine, berries, and peanuts, is a natural product with well-known favorable effects on endothelial NO availability in the vasculature [3,4]. It has been shown that resveratrol promotes endothelial function by increasing eNOS expression, by enhancing eNOS activity [5]. Resveratrol has been reported to increase endothelial NO release via short and long-term effects. The rapid effect of resveratrol includes the phosphorylation of eNOS by AMPK, or ERK 1 and 2, and the deacetylation of eNOS by SIRT1 [6,7,8,9]. Long-term resveratrol increases eNOS mRNA and protein expression [10,11,12,13]. Resveratrol supplementation has been shown to be beneficial for endothelial function in several animal models [14,15]. However, an increase in eNOS expression upon resveratrol treatment could not be reproduced *in vivo* [15,16]. Apart from its cardiovascular effects, resveratrol supplementation was suggested to be beneficial for combating a wide range of diseases and conditions including inflammatory disorders, cancer, Alzheimer’s disease and aging. Multiple cellular targets have been proposed to be responsible for the action of resveratrol [3,4,12].

In the last decades, many studies have been performed to elucidate the mechanism of action of resveratrol, with the aim to combine results obtained from cell culture systems with the data from *in vivo* observations. However, the relevance of *in vitro* data for the interpretation of *in vivo* effects of resveratrol is controversial since the oral bioavailability of resveratrol *in vivo* is rather limited due to its metabolization to resveratrol sulfates and glucuronides [17,18,19]. This sparked the issue whether resveratrol is in fact the active molecule *in vivo*. The oral intake of a dietary relevant dose of 25 mg of resveratrol was reported to lead to a plasma concentration of resveratrol in the nanomolar range, whereas the concentration of metabolites in plasma was in the micromolar range [20]. Additionally, the significant effects of resveratrol in *in vivo* experiments suggest that some resveratrol metabolites could be biologically relevant [21], either directly or by back conversion to resveratrol in target cells via glucuronidases and sulfatases [22].

Resveratrol metabolites have been reported to elicit a wide range of bioactivities similar to their parent compound resveratrol. For instance, resveratrol-4'-glucuronide, resveratrol-3-glucuronide, and resveratrol-3-sulfate reduced fat accumulation in adipocytes and influenced adipokine expression and secretion. Hence, they may be involved in anti-obesity effects after resveratrol consumption [23,24]. Resveratrol-3-sulfate and resveratrol disulfates showed anti-inflammatory activity by counteracting an inflammatory challenge in macrophages [25]. Furthermore, Resveratrol-4'-sulfate inhibited cyclooxygenase-1 and 2 (COX-1 and COX-2) with a potency similar to resveratrol, whereas resveratrol-3-sulfate and resveratrol-3-glucuronide were only weakly active on these enzymes [19]. Additionally, resveratrol-3-sulfate and resveratrol-4'-sulfate were able to increase sirtuin-1 activity to the same extent as resveratrol [26]. Sulfate and glucuronide metabolites were reported to inhibit colon cancer cell growth [22,27,28]. Resveratrol metabolites are therefore likely to be involved in the anti-cancer effects of resveratrol.

To interpret and understand results from *in vivo* studies, it is necessary to identify relevant resveratrol metabolites and to study their mode of action.

In this study, we determine the changes in eNOS activity and endothelial NO release as well as intracellular ROS levels, upon exposure of endothelial cells to several physiological resveratrol metabolites.

## 2. Results and Discussion

### 2.1. Impact of Resveratrol and Its Metabolites on eNOS Enzyme Activity

Since none of the metabolites could so far be identified as the major active compound, it is important to determine the activity of the physiologically relevant metabolites for every function and target, as in this study for the endothelial NO system.

Several *in vitro* studies reported beneficial effects of resveratrol on the eNOS system in micromolar [8,10,11,29] as well as in nanomolar concentrations [7,13,30]. Micromolar concentrations of resveratrol have been reported to elevate eNOS mRNA levels, activity and expression levels [8,10,11,29]. Additionally, an increase in eNOS expression was observed upon administration of 100 nM resveratrol [13]. A study performed by Klinge et al could demonstrate that nanomolar resveratrol induces ERα-Cav-1-c-SRC interaction, resulting in NO production through a Gα-protein-coupled mechanism, suggesting an explanation for the effects seen with resveratrol *in vivo* [30].

For this study we decided to test resveratrol metabolites in micromolar concentrations since oral administration of 5 g of resveratrol led to a plasma concentration of metabolites in this concentration range [31]. The parent compound resveratrol itself was used in the same concentrations as previously as a control [10,29], although most likely physiologically not relevant.

**Figure 1:**
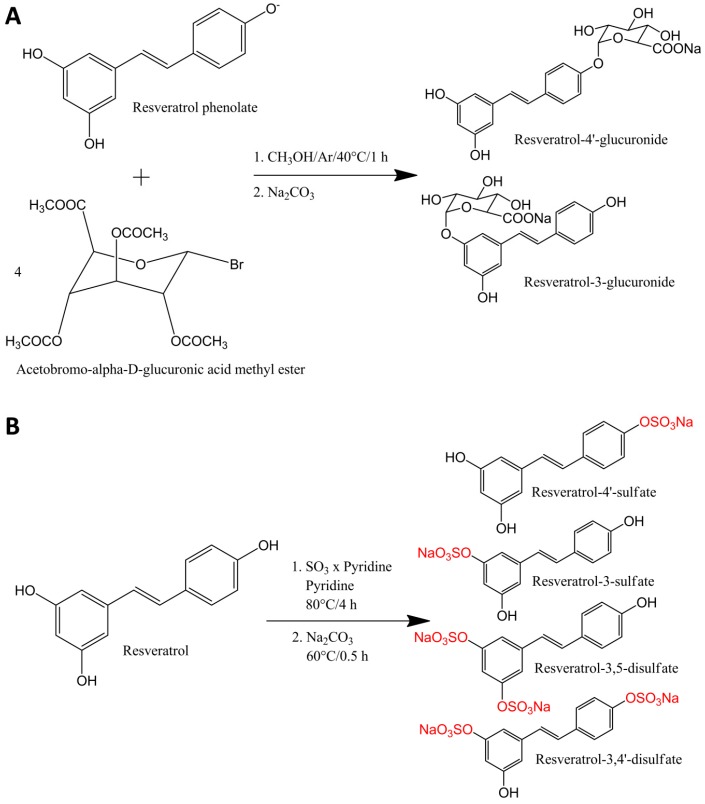
Synthesis of resveratrol glucuronides (**A**) and sulfates (**B**).

It has been previously reported that resveratrol is able to enhance eNOS activity, among others, by stimulation of eNOS expression. This is achieved via stabilization of eNOS mRNA and direct increase in eNOS gene transcription [10]. Since effects on gene transcription are rather slow, we determined eNOS enzyme activity and endothelial NO release 24 h after treating endothelial EA.hy926 cells with resveratrol or resveratrol metabolites (Figure 1).

**Figure 2:**
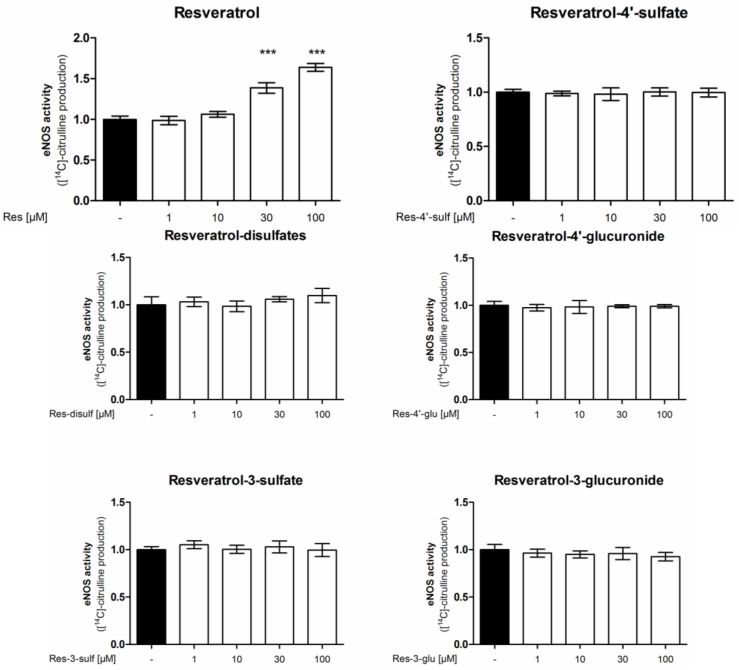
Influence of resveratrol and its metabolites on eNOS enzyme activity. EA.hy926 cells were treated with the indicated concentrations of resveratrol or its metabolites for 24 h. Then an [^14^C]l-arginine/[^14^C]l-citrulline conversion assay was performed as described. [^14^C]l-citrulline production was normalized to the untreated control (*** *p* < 0.001; mean ± SD, *n* = 3).

In a first experiment the [^14^C]l-arginine/[^14^C]l-citrulline conversion assay was utilized to measure eNOS enzyme activity (Figure 2). Treatment of endothelial cells with resveratrol concentration-dependently increased eNOS enzyme activity up to 1.64 fold at 100 µM compared to control conditions, thereby confirming the previously reported eNOS stimulation by resveratrol [10]. As resveratrol is metabolized *in vivo*, we studied the effect of metabolites on eNOS activity. We used resveratrol-3-sulfate, resveratrol-4'-sulfate, resveratrol disulfates, resveratrol-3-glucuronide and resveratrol-4'-glucuronide in concentrations from 1 to 100 µM in the same assay. However, none of the tested resveratrol sulfate or glucuronide metabolites was able to elicit a significant change in eNOS enzyme activity.

**Figure 3:**
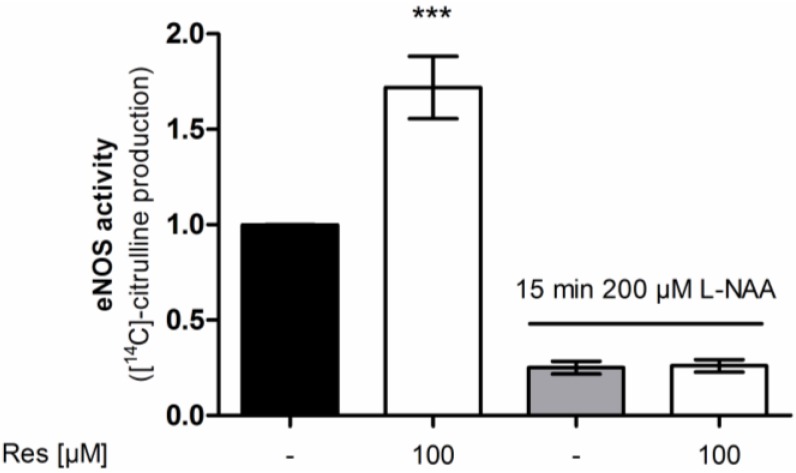
Influence of the NOS inhibitor l-NAA on the effect of resveratrol on eNOS enzyme activity. EA.hy926 cells were treated with the indicated concentrations of resveratrol for 24 h. Then an [^14^C]l-arginine/[^14^C]l-citrulline conversion assay was performed as described both in the presence and absence of the NOS inhibitor l-NAA. [^14^C]l-citrulline production was normalized to the untreated control (*** *p* < 0.001; mean ± SD, *n* = 3).

To demonstrate that [^14^C]l-citrulline production upon resveratrol treatment derives from eNOS enzyme activity the experiment was repeated in the presence of the NOS-inhibitor l-NAA (N^G^-Amino-l-arginine hydrochloride) (Figure 3). l-NAA reduced the basal and completely blocked the resveratrol-enhanced arginine conversion thereby confirming the NOS-specificity of the resveratrol effect.

### 2.2. Impact of Resveratrol and Its Metabolites on Endothelial NO Release

**Figure 4:**
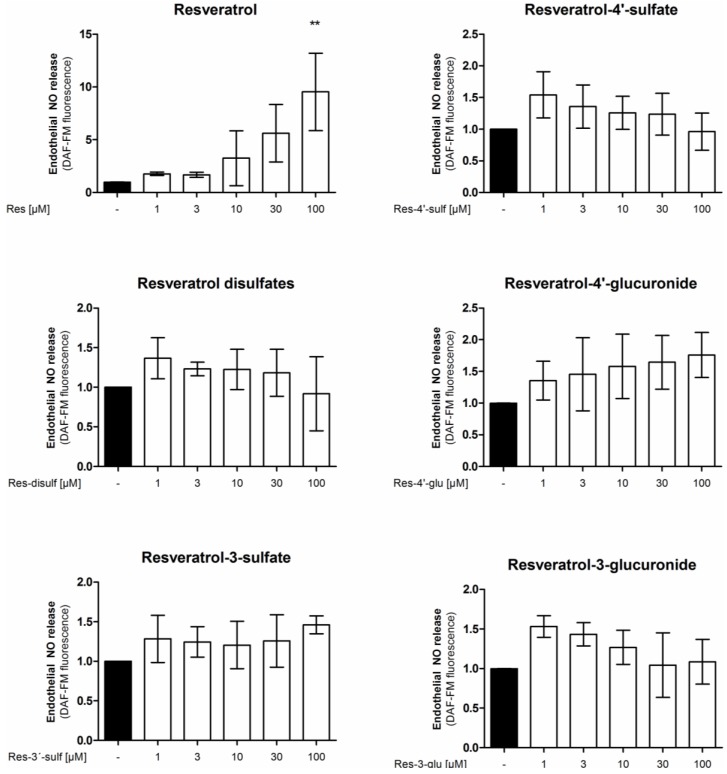
Influence of resveratrol and its metabolites on endothelial NO release. EA.hy926 cells were treated with the indicated concentrations of compounds for 24 h. Endothelial NO release was measured by incubation with the NO-sensitive fluorescent probe diaminofluorescein-FM. Fluorescence values were normalized to the number of viable cells as determined by resazurin staining (** *p* < 0.01; mean ± SD, *n* = 3).

It is possible that the tested resveratrol metabolites are not directly changing eNOS enzyme activity but enhance the bioavailability of endothelial NO for example through the inhibition of superoxide-mediated NO inactivation [5]. Therefore, we determined the levels of endothelial NO release in EA.hy926 cells after treatment with resveratrol or resveratrol sulfate or glucuronide conjugates for 24 h (Figure 4). Resveratrol concentration-dependently increased endothelial NO release reaching significance and displaying a 5-fold increase at 30 µM. However, none of the tested resveratrol sulfate or glucuronide metabolites was able to elicit a significant change in endothelial NO release.

### 2.3. Impact of Resveratrol and Its Metabolites on Intracellular Reactive Oxygen Species (ROS) Levels

Increased ROS levels have been found to be a major contributor to the development of cardiovascular diseases. Resveratrol is well-known to reduce ROS levels in endothelial cells. It not only directly scavenges ROS [32], but also activates expression of antioxidant enzymes such as superoxide dismutase 1, glutathione peroxidase or thioredoxin [33,34]. Under certain conditions resveratrol can, however, elicit pro-oxidant effects on the endothelium, suggesting a dual role of resveratrol in endothelial cells [35,36]. In a recent study 10 µM doses of resveratrol were shown to exert pro-oxidant effects whereas concentrations as low as 0.5 µM had an opposite effect [35]. Apart from the applied doses, the oxidative state of the cells seems to be important. Resveratrol has been shown to act as a pro-oxidant under low oxidative conditions and turning into an antioxidant under strong oxidative conditions [37].

**Figure 5:**
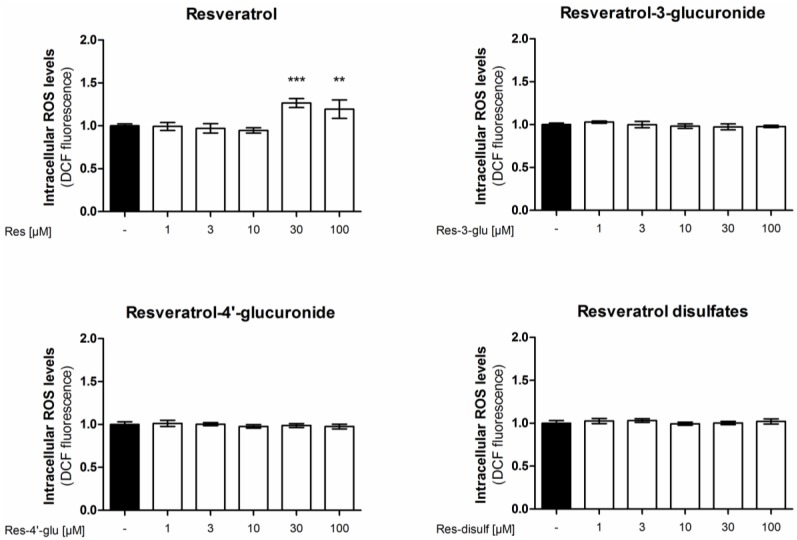
Influence of resveratrol and its metabolites on intracellular reactive oxygen species levels. EA.hy926 cells were treated with the indicated concentrations of resveratrol or its metabolites for 24 h. Intracellular ROS was measured by incubation with the fluorescent probe 2',7'-dichloro-fluorescein-diacetate (DCF). (** *p* < 0.01, *** *p* < 0.001; mean ± SD, *n* = 4).

To elucidate if the tested resveratrol metabolites affect the cellular ROS detoxification systems, we investigated their effect on intracellular ROS levels. We treated EA.hy926 cells with resveratrol or resveratrol sulfate or glucuronide derivatives for 24 h and determined their ROS load by DCF staining (Figure 5). However, apart from the parent compound resveratrol none of the tested resveratrol metabolites was able to significantly change intracellular ROS levels. The fact that resveratrol, starting at 30 µM, increased intracellular ROS levels is in agreement with an already published study [35].

## 3. Experimental Section

### 3.1. Chemicals and Cell Culture Reagents

Dulbecco’s modified Eagle’s medium (DMEM) without phenol red containing 4.5 g/L glucose, glutamine, benzylpenicillin and streptomycin were purchased from Lonza (Verviers, Belgium), HAT supplement (100 μM hypoxanthine, 0.4 μM aminopterin and 16 μM thymidine) from Biochrom (Berlin, Germany), and trypsin from Cambrex (Verviers, Belgium). Fetal bovine serum (FBS) was obtained from Gibco via Invitrogen (Paisley, UK). A23187 were bought from Alexis Biochemicals (Lausen, Switzerland) and [^14^C]l-arginine (346 mCi/mmol) from New England Nuclear (Boston, MA, USA). N^G^-Amino-l-arginine (hydrochloride) (l-NAA) was obtained from Cayman Chemicals via Biomol (Hamburg, Germany). All other chemicals, including trans-resveratrol, were bought from Sigma-Aldrich (Vienna, Austria). TLC plates were bought from Machery-Nagel (Markus Bruckner Analysentechnik, Linz, Austria).

### 3.2. Synthesis and Purification of Resveratrol Conjugates

Synthesis of resveratrol glucuronides (Figure 1A) was performed as described previously [18]. Synthesis and purification of resveratrol sulfates (Figure 1B) were modified according to Wenzel *et al.* [18]. Briefly, 4 mmol trans-resveratrol and 8 mmol sulfur trioxide pyridine complex were separately dissolved in anhydrous pyridine. The reaction was started by slowly adding resveratrol to sulfur trioxide pyridine complex. The mixture was kept under nitrogen atmosphere and was heated up to 80 °C under reflux for 4 h. To convert the synthesis products into sodium salts, 160 mmol Na_2_CO_3_ was added to the mixture and stirred at 60 °C for another 30 min. Prior to purification of the resveratrol sulfates, the solvent was removed by rotary evaporation. The purification of the resveratrol sulfates was performed by a Dionex Ultimate 3000 semi-preparative LC system (Thermo Fisher Scientific, Vienna, Austria) equipped with a UV detector set to 300 nm. The resveratrol sulfates were separated at a flow of 2.5 mL/min on a RP18 column (Synergi Fusion, 250 × 10 mm, 4 µm, Phenomenex, Aschaffenburg, Germany) using a methanol gradient. The gradient started with 3% methanol and 97% ammonium formate (10 mM, pH 8.2) and reached 75% methanol after 45 min before 100% methanol was reached after another 2 min. To separate resveratrol-3-sulfate from resveratrol-4'-sulfate, the corresponding fraction from the first purification was further purified using the same column and mobile phase. The second purification step started with 25% methanol, which was kept constant for 35 min, and ended with 100% methanol after 40 min. After structure identification, purities of 91.5%, 98.4% and 98.6%, for resveratrol-3-sulfate, resveratrol-4'-sulfate and resveratrol disulfates (resveratrol-3,4'-disulfate:resveratrol-3,5-disulfate, 1:1) were determined by means of LCMS(+) and 2D-NMR, as described earlier [18].

### 3.3. Cell Culture

The human endothelial cell line EA.hy926 (kindly provided by Dr. C.-J.S. Edgell, University of North Carolina, Chapel Hill, NC, USA) [38] was grown in DMEM (without phenol red) supplemented with 2 mM glutamine, 100 U/mL benzylpenicillin, 100 μg/mL streptomycin, HAT supplement, and 10% FBS up to passage 26. Resveratrol and resveratrol metabolites were dissolved in dimethyl sulfoxide (DMSO) and stored at −80 °C. Final DMSO concentrations did not exceed 0.1%. Control cells were always treated with an equal volume of solvent.

### 3.4. [^14^C]l-Arginine/[^14^C]l-Citrulline Conversion Assay

The enzymatic reaction catalyzed by eNOS converts the amino acid arginine into citrulline and NO. [^14^C]l-citrulline production can thus serve as a surrogate marker of NO production. The assay was performed as previously described [39]. In short, EA.hy926 cells were seeded in six-well plates at a density of 5 × 10^5^ cells/well and treated with test compounds at confluence, after approximately 72 h. Then the endothelial cells were equilibrated in HEPES buffer (HEPES 10 mM, NaCl 145 mM, KCl 5 mM, MgSO_4_ 2 mM, α-d(+)-Glucose 10 mM, CaCl_2_ × 2H_2_O 1.5 mM, pH 7.4) for 10 min at 37 °C. In some experiments 200 µM of the irreversible eNOS inhibitor N^G^-Amino-l-arginine (hydrochloride) (l-NAA) was added and the buffer was replaced after 15 min. Subsequently, 0.32 μM [^14^C]l-arginine (346 mCi/mmol) and 1 μM of the calcium ionophore A23187 were added for 15 min. The reaction was stopped by lysing cells, followed by extraction with ethanol and ethanol/water. The extracts were dried under vacuum (SPD 1010 SpeedVac, Thermo Savant, Thermo Scientific, Langenselbold, Germany) and resolved in water/methanol (1:1). After separation of [^14^C]l-arginine from [^14^C]l-citrulline by thin layer chromatography (Polygram SIL N-HR, Machery-Nagel, Austria) in the solvent system water:chloroform:methanol:ammonium hydroxide 25% (2:1:9:4, v/v/v/v), [^14^C]l-citrulline was quantified by autoradiography in a phosphoimager (BAS-1800II, Fujifilm, Düsseldorf, Germany). AIDA software (raytest, Langenzersdorf, Austria) was used for densitometric analysis.

### 3.5. Quantification of NO Release by Diaminofluorescein-FM (DAF-FM)

Quantification of NO released from endothelial cells was performed using diaminofluorescein-FM (DAF-FM), a NO-sensitive fluorescent probe. EA.hy926 cells were seeded in 96-well plates at a density of 2.5 × 10^4^ cells/well and were treated with test compounds at confluence after approximately 72 h. Cells were washed two times with PBS + (137 mM NaCl, 2.68 mM KCl, 8.1 mM Na_2_HPO_4_, 1.47 mM KH_2_PO_4_, 0.5 mM MgCl_2_ × 6H_2_O, 0.68 mM CaCl_2_ × 2H_2_O) containing 100 μM arginine and equilibrated 10 min in this buffer. Then, A23187 was added to a final concentration of 1 μM and DAF-2 to a final concentration of 0.1 μM, and the cells were incubated for 1 h at 37 °C. Addition of l-NAME to a final concentration of 200 μM allowed correction for non-NO-specific fluorescence. The supernatant was transferred to a black 96-well plate and fluorescence was measured in a plate reader (Genios Pro, Tecan, Grödig, Austria) with an excitation wavelength of 485 nm and an emission wavelength of 520 nm. Fluorescence values were normalized to viable cells as determined by resazurin conversion method [40]. For this, the cells were incubated with 0.1 mg/mL resazurin in PBS for 30 min before measuring the fluorescence in a plate reader (Genios Pro, Tecan, Grödig, Austria) at an excitation wavelength of 535 nm and an emission wavelength of 590 nm.

### 3.6. Measurement of Intracellular Reactive Oxygen Species (ROS) Levels 

Intracellular ROS levels were measured with the 2',7'-dichloro-fluorescein-diacetate (DCF) probe. EA.hy926 cells were seeded in 12-well plates at a density of 1.6 × 10^5^ cells/well and were treated with test compounds at confluence after approximately 72 h. Catalase (activity 21600 U/mg protein) was added to the cell culture medium at 50 U/mL and co-incubated with the test substances to inhibit formation of ROS in the cell culture supernatant [41]. After 24 h treatment, cells were washed once with HBSS (140 mM NaCl, 5 mM KCl, 0.14 mM Na_2_HPO_4_, 0.37 mM KH_2_PO_4_, 0.80 mM MgSO_4_, 1.2 mM CaCl_2_ × 2H_2_O, 5.6 mM glucose, 20 mM HEPES) and incubated with 20 µM DCF in HBSS for 25 min at 37 °C. After washing the cells once with HBSS, cells were trypsinized and resuspended in PBS containing 2% BSA. Samples were measured at a flow cytometer FACS Calibur^TM^ (BD Biosciences, Franklin Lakes, NJ, USA) at the excitation/emission wavelength 488/530 nm (channel FL-1). Geometric means were calculated using CellQuest^TM^ Pro.

### 3.7. Statistical Methods 

Statistical analysis was done using GraphPad Prism software version 4.03 (GraphPad Software Inc., La Jolla, CA, USA). To determine statistical significance one-way analysis of variance (ANOVA) with Bonferroni post-test was performed. Asterisks indicate statistically significant differences (* *p* < 0.05; ** *p* < 0.01; *** *p* < 0.001). Figures with bar graphs represent mean ± SD of at least three independent experiments.

## 4. Conclusions 

To the best of our knowledge this study shows for the first time the absence of a direct role of the main physiological resveratrol metabolites on eNOS enzyme activity, NO release and intracellular ROS production in endothelial cells. Further studies are necessary to evaluate a possible influence of resveratrol metabolites not tested here, such as dihydro-resveratrol and its glucuronic and sulfated derivatives, which have recently been identified in rat liver and adipose tissue [42] and the potential synergy of combinations of resveratrol and the single metabolites. Back-conversion of resveratrol metabolites to resveratrol in the target tissue would also account for the lack of activity of metabolites observed in this study. Another explanation for the lack of activity of the resveratrol metabolites could be that endothelial function is influenced by a here not investigated pathway, or that the applied *in vitro* system is too artificial for the complex situation *in vivo*.
